# IRE1α inhibition decreased TXNIP/NLRP3 inflammasome activation through miR-17-5p after neonatal hypoxic–ischemic brain injury in rats

**DOI:** 10.1186/s12974-018-1077-9

**Published:** 2018-02-02

**Authors:** Di Chen, Brandon J. Dixon, Desislava M. Doycheva, Bo Li, Yang Zhang, Qin Hu, Yue He, Zongduo Guo, Derek Nowrangi, Jerry Flores, Valery Filippov, John H. Zhang, Jiping Tang

**Affiliations:** 1 Cerebrovascular Diseases Laboratory, Institute of Neuroscience, Chongqing Medical University, Chongqing, 400016 China; 2 Department of Basic Sciences, Loma Linda University School of Medicine, Loma Linda, CA 92350 USA; 3 Department of Neurosurgery, Loma Linda University School of Medicine, Loma Linda, CA 92350 USA

**Keywords:** Hypoxia–ischemia, IRE1α, MicroRNA-17 (miR-17), Neonatal, Nod-like receptor protein 3 (NLRP3), Thioredoxin-interacting protein (TXNIP)

## Abstract

**Background:**

The endoplasmic reticulum (ER) is responsible for the control of correct protein folding and protein function which is crucial for cell survival. However, under pathological conditions, such as hypoxia–ischemia (HI), there is an accumulation of unfolded proteins thereby triggering the unfolded protein response (UPR) and causing ER stress which is associated with activation of several stress sensor signaling pathways, one of them being the inositol requiring enzyme-1 alpha (IRE1α) signaling pathway. The UPR is regarded as a potential contributor to neuronal cell death and inflammation after HI. In the present study, we sought to investigate whether microRNA-17 (miR-17), a potential IRE1α ribonuclease (RNase) substrate, arbitrates downregulation of thioredoxin-interacting protein (TXNIP) and consequent NLRP3 inflammasome activation in the immature brain after HI injury and whether inhibition of IRE1α may attenuate inflammation via miR-17/TXNIP regulation.

**Methods:**

Postnatal day 10 rat pups (*n* = 287) were subjected to unilateral carotid artery ligation followed by 2.5 h of hypoxia (8% O_2_). STF-083010, an IRE1α RNase inhibitor, was intranasally delivered at 1 h post-HI or followed by an additional one administration per day for 2 days. MiR-17-5p mimic or anti-miR-17-5p inhibitor was injected intracerebroventricularly at 48 h before HI. Infarct volume and body weight were used to evaluate the short-term effects while brain weight, gross and microscopic brain tissue morphologies, and neurobehavioral tests were conducted for the long-term evaluation. Western blots, immunofluorescence staining, reverse transcription quantitative real-time polymerase chain reaction (RT-qPCR), and co-immunoprecipitation (Co-IP) were used for mechanism studies.

**Results:**

Endogenous phosphorylated IRE1α expression was significantly increased after HI. Intranasal administration of STF-083010 alleviated brain injury and improved neurological behavior. MiR-17-5p expression was reduced after HI, and this decrease was attenuated by STF-083010 treatment. MiR-17-5p mimic administration ameliorated TXNIP expression, NLRP3 inflammasome activation, caspase-1 cleavage, and IL-1β production, as well as brain infarct volume. Conversely, anti-miR-17-5p inhibitor reversed IRE1α inhibition-induced decrease in TXNIP expression and inflammasome activation, as well as exacerbated brain injury after HI.

**Conclusions:**

IRE1a-induced UPR pathway may contribute to inflammatory activation and brain injury following neonatal HI. IRE1a activation, through decay of miR-17-5p, elevated TXNIP expression to activate NLRP3 inflammasome and aggravated brain damage.

**Electronic supplementary material:**

The online version of this article (10.1186/s12974-018-1077-9) contains supplementary material, which is available to authorized users.

## Background

Cerebral hypoxia–ischemia (HI) is a principal risk factor of perinatal brain injuries in both full-term and preterm neonates worldwide leading to acute mortality and chronic disability [1–4]. Despite current therapeutic modalities, HI still accounts for 23% of all neonatal deaths globally [5]. Survivors of perinatal asphyxia suffer lifelong disabilities such as cerebral palsy, epilepsy, and cognitive, behavioral, attentional, socialization, and learning difficulties [6–10]. Although numerous neuroprotective treatments have appeared promising in animal experiments, most of them were not reliable or effective in human patients with hypoxic ischemic encephalopathy (HIE). Thus, there is an urgent need for the identification of new therapeutic targets for salvaging brain to address treatment of HIE.

It is now well established that HI brain injury is a progressive and evolving process, and multiple biochemical mechanisms and pathways contribute to both early and delayed injury [11]. Mounting evidence has revealed that endoplasmic reticulum (ER) stress is involved in initiating cell death after HI insult [12, 13]. The ER is a well-orchestrated protein-folding center; thus, maintenance of ER homeostasis is imperative for cellular functions. Many physiological or pathological factors, such as hypoxia, acidosis, and depletion of ER calcium stores, can disturb ER homeostasis and thus negatively impact on protein folding processes leading to an accumulation of unfolded or misfolded proteins. Elevated levels of these unfolded or misfolded proteins within the ER lumen trigger a configuration commonly named ER stress. The ER stress is monitored by three transmembrane receptors named respectively inositol-requiring enzyme 1 (IRE1), protein kinase-like endoplasmic reticulum kinase (PERK), and activating transcription factor 6 (ATF6) [14]. To alleviate the stress and restore normal cell function, a highly conserved adaptive mechanism referred to as the unfolded protein response (UPR), induced by the above sensors, has evolved to reduce the amount of misfolded proteins in the lumen of this organelle [15, 16]. However, under prolonged and irremediable ER stress, the UPR signaling pathway switches from pro-survival to pro-apoptotic, committing the cells to death [17, 18]. For this reason, the UPR is considered as the crossroads of cellular life and death during ER stress.

Thioredoxin-interacting protein (TXNIP) is an endogenous inhibitor of thioredoxin (TRX), a major cellular thiol-reducing and antioxidant protein. TXNIP was also identified as a Nod-like receptor protein 3 (NLRP3) binding protein, where association between the two proteins was necessary for subsequent inflammasome formation and activation [19]. It has been established that TXNIP dissociates from TRX and associates with NLRP3 under oxidative stress [20–23]. Subsequently, activation of NLRP3 inflammasome results in pro-caspase-1 cleavage and IL-1β secretion, thereby increasing cell death. Therefore, TXNIP is a molecular connection between UPR and inflammation and serves as a switch that redirects cell fate from an adaptive to terminal UPR [24]. Considering that TXNIP plays critical role in flipping the switch to control cell fate, it is of great significance to identify those regulators of TXNIP under ER stress conditions. The rapid upregulation of TXNIP upon ER stress is due, in part, to changes in stability of TXNIP mRNA. Under resting conditions, TXNIP mRNA is inherently labile and its half-life is short, but it becomes significantly stabilized during ER stress [25].

MicroRNAs (miRNAs), a type of small non-protein-coding mRNAs containing about 22 nucleotides, are implicated in negative regulation of gene expression post transcription through binding 3′-untranslated regions (3′-UTR) of target genes and controlling their translation and/or degradation [26–28]. Increasing evidence supports that miRNA dysfunction is a contributing factor for many central nervous system pathologies including stroke [29–31]. Recently, miRNAs have emerged as key regulators of ER homeostasis and important players in UPR-dependent signaling pathways. MicroRNA-17 (miR-17) is a member of miR-17-92 cluster, located on the human chromosome 13 and on the mouse chromosome 14 [32]. This cluster is the first group of miRNAs to be implicated in a developmental syndrome and indispensable for proliferation of multiple tissues. Moreover, a large body of literature has revealed that the miR-17-92 cluster is also involved in tumorigenicity and other diseases [32]. It has been confirmed that miR-17 is a regulator of TXNIP mRNA stability. There are highly conserved seed sequences for miR-17 in the TXNIP 3′-UTR which are found to govern TXNIP mRNA expression at posttranscriptional level. Oxidative stress can disrupt ER homeostasis, thereby activating the UPR. Under irremediable ER stress, ribonuclease activity of IRE1α, one of the three sensors of UPR, initiates degeneration of miR-17, subsequently TXNIP mRNA becomes more stable to elevate TXNIP expression levels [28].

Based on previous studies, we hypothesized that IRE1α activation induces increased TXNIP level through decaying miR-17 to activate NLRP3 inflammasome thus deteriorating brain injury following neonatal HI. In the present study, we sought to investigate the potential role of IRE1α/miR-17/TXNIP pathway in inflammasome activation and brain injury after HI insult and explore the potential therapeutic utility of IRE1α inhibitor in neonatal HI model.

## Methods

### Animals

All procedures for this study were approved by the Institutional Animal Care and Use Committee (IACUC) of Loma Linda University and were in accordance with the National Institutes of Health guidelines for the treatment of animals. A total of 287 postnatal day 10 (P10) Sprague–Dawley rat pups (weighing 13~20 g) were used, of which 9 died while in the hypoxia chamber and 10 died after HI insult and were excluded in this study. Animals of both genders were used.

### Neonatal hypoxia–ischemia exposure model

A modified Rice–Vannucci model [33] was used as previously described [34, 35]. Briefly, P10 unsexed rat pups (Harlan Laboratories, Indianapolis, IN) were anesthetized with 3% isoflurane and maintained at 2.5% isoflurane in air during surgery. Following aseptic preparation, a longitudinal midline incision was made in the anterior neck. After the right common carotid artery was identified and isolated, it was double ligated using 5–0 surgical suture and transected between the ligatures. Total surgery time was controlled to be less than 5 min to minimize the standard deviation [36].

Pups were left to recover for 1 h after surgery and then placed in a hypoxia chamber (perfused with 8% O_2_/92% N_2_) kept in a water bath maintained at 37 °C for 2.5 h and then returned to their dams. The sham group of rat pups had the right common carotid artery exposed, but not ligated or transected, and the animals were not exposed to hypoxic conditions.

### Experimental design

The experiment was designed as follows.

#### Experiment I

To evaluate expression level of endogenous phosphorylated IRE1α, time course experiment was conducted at 0, 3, 6, 12, 24, and 72 h after HI (*n* = 5/time point), with samples from the right/ipsilateral hemisphere, using western blot.

In addition, to determine pIRE1α expression on different cell types, double immunohistochemistry staining of pIRE1α with either NeuN (neuronal marker), Iba-1 (microglial marker), or GFAP (astrocyte marker) was performed in sham and 6 h after HI (*n* = 3/group).

#### Experiment II

To evaluate short-term outcome of IRE1α inhibition, pups were randomly divided into five groups: sham (*n* = 12), HI (*n* = 12), vehicle+HI (*n* = 14), STF-15+HI (STF-083010 15 μg/pup, *n* = 6), and STF-45+HI (STF-083010 45 μg/pup, *n* = 13). An IRE1α inhibitor STF-083010 was intranasally administered at 1 h after HI or at 1, 24, and 48 h after HI (three total administrations). TTC staining and body weight were performed at 24 or 72 h post HI.

#### Experiment III

To assess long-term outcomes of IRE1α inhibition, pups were randomly divided into three groups: sham (*n* = 8), vehicle+HI (*n* = 11), and STF+HI (*n* = 11). STF-083010 (45 μg/pup) was intranasally administered at 1 h after HI. Neurobehavioral tests, evaluation of systemic organ weight, brain weight, and brain morphology were performed at 5 or 6 weeks after HI.

#### Experiment IV

To explore miR-17-5p expression change in response to HI, reverse transcription quantitative real-time polymerase chain reaction (RT-qPCR) for miR-17-5p quantitation was conducted in ipsilateral/right hemisphere of each group at 3, 6, and 24 after HI insult (*n* = 4/time point).

To evaluate the effect of IRE1α inhibition on miR-17-5p expression level, pups were randomly divided into three groups: sham (n = 4), vehicle+HI (*n* = 8), and STF+HI (*n* = 8). miRNA quantitation was performed at 6 or 24 h post HI.

#### Experiment V

To choose effective dose of miR-17-5p mimic or inhibitor and assess the effect of miR-17-5p on TXNIP expression, negative control or Syn-rno-miR-17-5p miScript miRNA mimic or Anti-rno-miR-17-5p miScript miRNA inhibitor were injected intracerebroventricularly. Pups were randomly divided into seven groups: naive, negative control for mimic, mir-17-5p mimic-0.05 (Syn-rno-miR-17-5p miScript miRNA mimic 0.05 nmol), mir-17-5p mimic-0.5 (Syn-rno-miR-17-5p miScript miRNA mimic 0.5 nmol), negative control for inhibitor, mir-17-5p inhibitor-0.1 (Anti-rno-miR-17-5p miScript miRNA inhibitor 0.1 nmol), and mir-17-5p inhibitor-1 (Anti-rno-miR-17-5p miScript miRNA inhibitor 1 nmol) (*n* = 6/group). Western blots and qPCR of ipsilateral/right hemisphere were conducted to detect TXNIP protein and mRNA expressions at 48 h after mimic or inhibitor administration in all groups.

#### Experiment VI

To determine the effects of miR-17-5p overexpression or inhibition on infarct area, TXNIP expression, NLRP3 inflammasome activation, and IL-1β production after HI, miR-17-5p mimic or inhibitor was intracerebroventricularly injected at 48 h before HI. Pups were randomly assigned into eight groups: sham, HI, negative control+HI, mir-17-5p mimic+HI, vehicle+HI, STF+HI, STF+negative control+HI, and STF+mir-17-5p inhibitor+HI (*n* = 12/group). TTC staining, western blots, and co-immunoprecipitation were performed to assess changes in infarct area, association between NLRP3 and TXNIP, cleaved caspase-1, and IL-1β expressions.

### Intranasal administration of drug

STF-083010 (Tocris Bioscienc, MN) was prepared as previously described with modification [37]. In brief, STF-083010 was dissolved in dimethyl sulfoxide (DMSO) as a stock solution and further diluted in 8% cremophor (cremophor in normal saline) immediately before administration. STF-083010 was administered intranasally in two doses of 15 and 45 μg/pup at 1 h after HI or at 1, 24, and 48 h after hypoxia (total of three administrations). The vehicle+HI group received DMSO diluted with 8% cremophor at the same volume as the treatment groups. Intranasal administration was performed as previously reported [38, 39]. The anesthetized pups were placed on their backs and administered either vehicle, STF-083010 (1.5 μg/μl), or STF-083010 (4.5 μg/μl) as nose drops (1 μl/drop) over a period of 20 min, alternating drops every 2 min between the left and right nares. A total volume of 10 μl was administered intranasally.

### Infarct volume measurement

Infarct volume was evaluated with 2,3,5-triphenyltetrazolium chloride monohydrate (TTC) (Sigma Aldrich, Inc., St Louis, MO USA) staining as previously described [40, 41]. Briefly, at 24 and 72 h after HI, pups were euthanized, and their brains were removed and sectioned into 2-mm slices using a rat brain matrix (Additional file 1). The brain sections were incubated in 2% TTC solution for 5 min in the dark, rinsed in phosphate-buffered saline (PBS), and then fixed in 10% formaldehyde. Brain infarct volume was traced and analyzed using ImageJ software (Version 1.43u; National Institutes of Health, Bethesda, MD, USA). The following formula was used to calculated percent infarct: [(total area of contralateral hemisphere) − (area of un-infarcted area of ipsilateral hemisphere)]/(total area of contralateral hemisphere × 2) for each slice.

### Neurobehavioral tests

The following neurobehavioral tests were performed in a blinded manner at 5 weeks (sensorimotor tests, T-Maze, foot fault, and rotarod tests) or 6 weeks (Morris water maze test) after HI insult.

#### Sensorimotor tests

Methodology was as previously described [42, 43] for the six sensorimotor tests and scored accordingly: 0 for immediate and correct placement; 1 for delayed and/or incomplete placement; and 2 for no placement. Scores corresponded to raw values: 0 score = 100; 1 score = 50; and 2 score = 0.

#### T-maze test

This is a test to ascertain short-term or working memory, as well as complex cortical function. The T-maze measured 40 (stem) × 46 (arm) × 10 (width) cm. Rats were placed in the stem of the maze in a dark environment and allowed to freely explore the two arms of the maze until they chose to turn into one of the arms. The sequence of left and right arm choices over 10 trials was expressed as the rate of spontaneous alternation [44].

#### Foot fault test

This is a test to assess motor coordination. Rats were placed onto an elevated horizontal wire grid floor for 2 min. The foot fault was defined as when the rat inaccurately placed a fore- or hindlimb, and it fell through one of the openings in the grid. All four limbs were observed for foot faults. The total number of left and right foot faults was recorded.

#### Rotarod test

Rotarod test is used to assess sensorimotor coordination. The apparatus (Columbus Instruments, Columbus, OH) consisted of a rotating horizontal cylinder (7 cm diameter × 9.5 cm wide) requiring continuous walking forward to avoid falling. Rats were placed on the cylinder initially at rest (stationary) for a maximum of 1 min. In the second round of testing, the cylinder was set in motion at a constant speed of 5 rotations per min (rpm) for a maximum of 1 min. In the third and fourth rounds of testing, the cylinder was respectively started at 5 and 10 rpm and accelerated by 2 rpm every 5 s. The latency to fall off the cylinder was detected and recorded [45].

#### Morris water maze test

The water maze test is used to assess the ability to learn spatial locations and memory. It was conducted at 6-week post HI injury as previously described [34]. Briefly, the rats need to find a visualized (cued test) or submerged (special test) platform in a pool of water with visual cues in the room. The water maze consisted of a metal pool (118 cm diameter) filled with water and a platform (22 cm diameter) that rats could step on to escape the water. The platform location and entry point were varied according to a preset scheme. For each trial, the rat was placed with its nose against the wall into the water at one of four release points and allowed to find the platform. All trials lasted a maximum of 60 s, at which point the rat was manually guided to the platform. All the activities were recorded, and the animals’ swimming paths were measured for quantification of distance, latency, and swimming speed by the Video Tracking System SMART-2000 (San Diego Instruments Inc., CA). Cued trials measured place learning with the platform visible above water. Spatial trials assessed spatial learning with the platform submerged and probe trials measured spatial memory once the platform was removed.

### Measurement of systemic organ weight

After removal of brain, the organs including heart, lungs, liver, spleen, and kidneys were harvested and weighed. Data for systemic organs were expressed as the ratio of organ weight to body weight, as previously described [46].

### Evaluation of brain damage

#### Brain weight

Hemispheric weight loss has been used as the primary variable to estimate brain damage in neonatal HI rats [44, 45, 47]. After Morris water maze test, the rats were euthanized under deep anesthesia and brains were removed, without prior perfusion. After the cerebellum and brain stem were dissected from the forebrain, the hemispheres were separated by a midline incision and weighed on a high-precision balance (sensitivity ± 0.001 g). The results were expressed as the mass ratio of ipsilateral (right) to contralateral (left) hemisphere, as previously described [47].

#### Evaluation of brain morphology

The brains were immersed in phosphate-buffered formalin (PBF) and were stored for 1 week to allow for adequate tissue saturation. The brains were then removed from PBF and immersed in a 30% sucrose solution until they settled at the bottom of the containers. The brains were cryoprotected and coronally sectioned into 10-μm-thick slices with a cryostat (Leica CM3050S-3-1-1, Bannockburn, IL). Nissl staining was performed as previously described [48]. For quantification of brain atrophy, the Nissl-stained coronal brain sections were photographed under light microscopy and analyzed using ImageJ software as previously described [49, 50]. The residual volume was presented as a volume percentage by the following formula: (ipsilateral volume/contralateral volume) × 100%.

### Western blotting

Western blotting was performed as described previously [51]. Under deep anesthesia, rats were perfused intracardially with 200 ml of ice-cold physiological saline solution (PBS). Brains were removed and immediately snap-frozen in liquid nitrogen and stored at − 80 °C until lysis. The brain was divided into right/ipsilateral and left/contralateral hemispheres, and only the right/ipsilateral hemisphere was homogenized by RIPA lysis buffer (sc-24948, Santa Cruz Biotechnology, Inc., TX, USA) with protease inhibitor cocktail and used for subsequent experiments for analysis of proteins. The insoluble material was removed by centrifugation at 14,000*g* at 4 °C for 20 min, and the supernatants were collected. Equal amounts of protein (50 μg) were loaded into a 10% sodium dodecyl sulfate–polyacrylamide (SDS-PAGE) gel. After being electrophoresed and transferred to a nitrocellulose membrane, the membrane was blocked with 5% non-fat blocking grade milk (Bio-Rad, Inc., Hercules, CA, USA) in Tween/Tris-buffered saline (TTBS) for 1 h at room temperature. The membranes were then incubated with the primary antibody overnight at 4 °C. The following primary antibodies were used: anti-IRE1α (1:1000), anti-pIRE1α (1:1000), anti-TXNIP (1:500), anti-cleaved caspase-1 (1:1000), anti-IL-1β (1:1000), and anti-NLRP3 (1:500). Nitrocellulose membranes were incubated with secondary antibodies (1:8000) for 1 h at room temperature. Immunoblots were then probed via ECL Plus chemiluminescence reagent kit (Amersham Bioscience, Arlington Heights, IL) followed by exposure to X-ray films. The immunoreactive bands were analyzed using ImageJ.

### Reverse transcription quantitative real-time polymerase chain reaction (RT-qPCR) for miRNA quantitation

Total RNA was isolated with TRIzol® (Invitrogen). Reverse transcription was performed using the miScript II RT kit (Qiagen). Equal amounts of total RNA (1 μg) were reverse-transcribed with 4 μl 5 × miScript HiSpec buffer, 2 μl 10 × miScript Nucleics Mix, and 2 μl miScript Reverse Transcriptase Mix at 37 °C for 60 min and 95 °C for 5 min. PCR reactions were then conducted using the miScript SYBR Green PCR kit (Qiagen). Each reaction contained 2 μl of the RT reaction product, 12.5 μl 2× QuantiTect SYBR Green PCR Master Mix, 2.5 μl 10 × miScript Universal Primer, and 2.5 μl 10× miScript Primer Assay in a total volume of 25 μl using the CFX96 Touch™ (Bio-Rad Laboratories, Inc.). The thermocycling program was set as follows: PCR initial activation at 95 °C for 15 min, 40 cycles of denaturation at 94 °C for 15 s, annealing at 55 °C for 30 s, and extension at 70 °C for 30 s. The quantification cycle data were collected using a CFX manager (Bio-Rad Laboratories, Inc.). Predesigned primer for miR-17-5p and U6 was from Qiagen (Table 1). The expression of miR-17-5p was normalized using U6 as the internal control. The relative starting quantity of each transcript was determined using the comparative CT method for relative quantification [52]. The PCR experiments were repeated four times, each using separate sets of samples.

**Table 1:** Materials purchased from companies with catalog numbers

Name	Catalog number	Company
Rn_miR-17-5p_1 miScript Primer Assay	MS00013118	Qiagen
Hs_RNU6-2_11 miScript Primer Assay	MS00033740	Qiagen
Syn-rno-miR-17-5p miScript miRNA Mimic	MSY0000786	Qiagen
Anti-rno-miR-17-5p miScript miRNA Inhibitor	MIN0000786	Qiagen
AllStars Negative Control siRNA	1,027,281	Qiagen
miScript Inhibitor Negative Control	1,027,272	Qiagen

### Intracerebroventricular infusion of miRNA mimic and inhibitor

miR-17-5p mimic, miR-17-5p inhibitor, and their negative controls were purchased from Qiagen; catalog numbers are listed in Table 1. Syn-rno-miR-17-5p miScript miRNA mimic or Anti-rno-miR-17-5p miScript miRNA inhibitor was injected intracerebroventricularly. Briefly, rat pups were fixed on a stereotaxic apparatus (Stoelting, Wood Dale, IL) under isoflurane inhalation (2%) at 48 h before HI. A scalp incision was made on the skull surface, and the bregma was exposed. miRNA mimic (0.05 or 0.5 nmol/pup) or inhibitor (0.1 or 1 nmol/pup) or their negative controls were injected with a 10-μL syringe (Hamilton, NV) at the location of 1.5 mm posterior and 1.5 mm lateral to the bregma and 1.7 mm below the dura in the ipsilateral hemisphere.

### Co-immunoprecipitation (IP) assay

A Pierce Co-IP Kit (Thermo Scientific) was used for examination of the change in association between TXNIP and NLRP3 in the ipsilateral hemisphere. The protocol followed the manufacturer’s guidelines as previously described [38]. Protein extracts were precipitated by an anti-TXNIP antibody, and then the precipitated protein was evaluated by western blotting using anti-NLRP3 antibody.

### Statistical analysis

All data are presented as means ± standard error of the mean (SEM). Statistical analysis was performed using SPSS (version 17.0; SPSS Inc., Chicago, IL, USA). Statistical differences were determined using the two-tailed Student’s *t* test for comparison of two groups or the one-way analysis of variance (ANOVA) followed by Student–Newman–Keuls (SNK) test for experiments with ≥ three groups. Water maze data was analyzed using the general linear models repeated measures analysis of variance. A value of *p* < 0.05 was considered significant.

We assigned six pups/group for most experiments (western blot and infarct area) to reach statistical significance as suggested by animal number power analysis. Long-term neurobehavior studies have *n* = 8/group. The distribution of animals for each endpoint is similar to our previous studies and was verified by sample size analysis using Sigmaplot (11.0). Sample sizes were calculated for all groups assuming a type I error (false positive) rate = 0.05 and power = 0.8 on a two-sided *t* test. Based on previous studies, expected mean values, and variation within groups, as well as the expected change in the means (a change of 30% for long-term advanced neurobehavioral analysis and 20% for western blotting), we concluded that a sample size of 6–8 pups/group are needed for the majority of the experiments.”

## Results

### Time course expression levels of endogenous phosphorylated IRE1α post HI

**Fig. 1:**
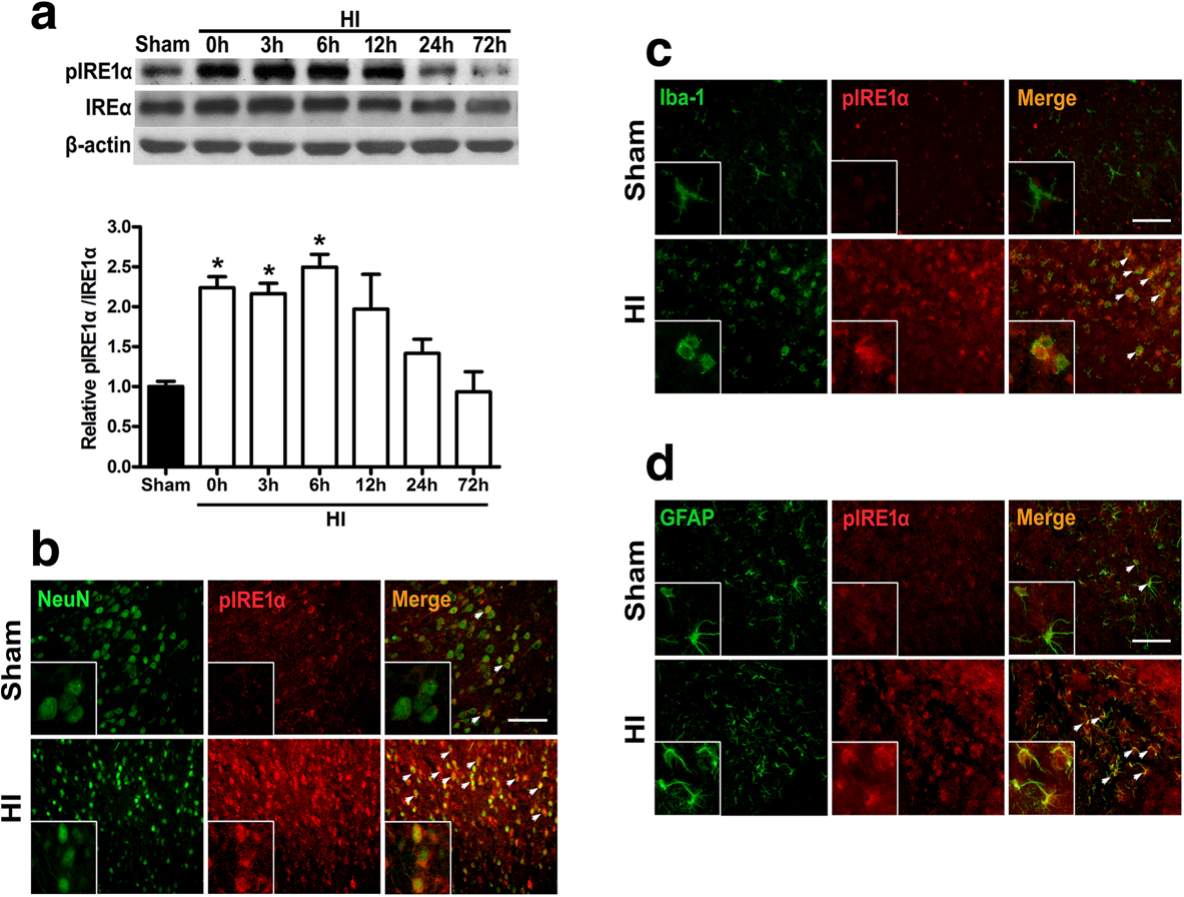
Time course expression of pIRE1α in brain tissues and Immunofluorescence staining showing co-localization post HI. **a** Western blot assay for temporal profile of pIRE1α and IRE1α expressions from ipsilateral hemisphere at 0, 3, 6, 12, 24, and 72 h post HI; data are expressed as mean ± SEM, *n* = 5 per group, per time point. Relative densities have been normalized against the sham group. *P < 0.05 compared with sham group. **b**–**d** Representative microphotographs of immunofluorescence staining for pIRE1α (red) co-localization on neurons (NeuN, green; **b**), microglia (Iba-1, green; **c**), and astrocytes (GFAP, green; **d**) at 6 h post HI. *n* = 3 per group, scale bar = 100 μm

To investigate whether IRE1α-mediated UPR post HI injury is activated, the temporal profile of phosphorylated IRE1α (pIRE1α) in the brain was measured by western blot (Fig. 1a). pIRE1α level in the ipsilateral hemisphere started to significantly increase as early as 0 h post HI and peaked at 6 h (*P* < 0.05 vs. sham). Although pIRE1α was sustained at a relatively high level until 12 h, there was no significant difference when compared to sham group. It returned to a level indistinguishable from sham by 24 h. Furthermore, in the frontal cortex region of the ipsilateral hemisphere at 6 h post HI injury, the pIRE1α immunoreactivity increased in neurons (pIRE1α/NeuN) (Fig. 1b), microglia (pIRE1α/Iba-1) (Fig. 1c), and astrocytes (pIRE1α/GFAP) (Fig. 1d).

### Intranasal administration of an IRE1α inhibitor (STF-083010) reduced brain infarct volume at 24 and 72 h post HI

**Fig. 2:**
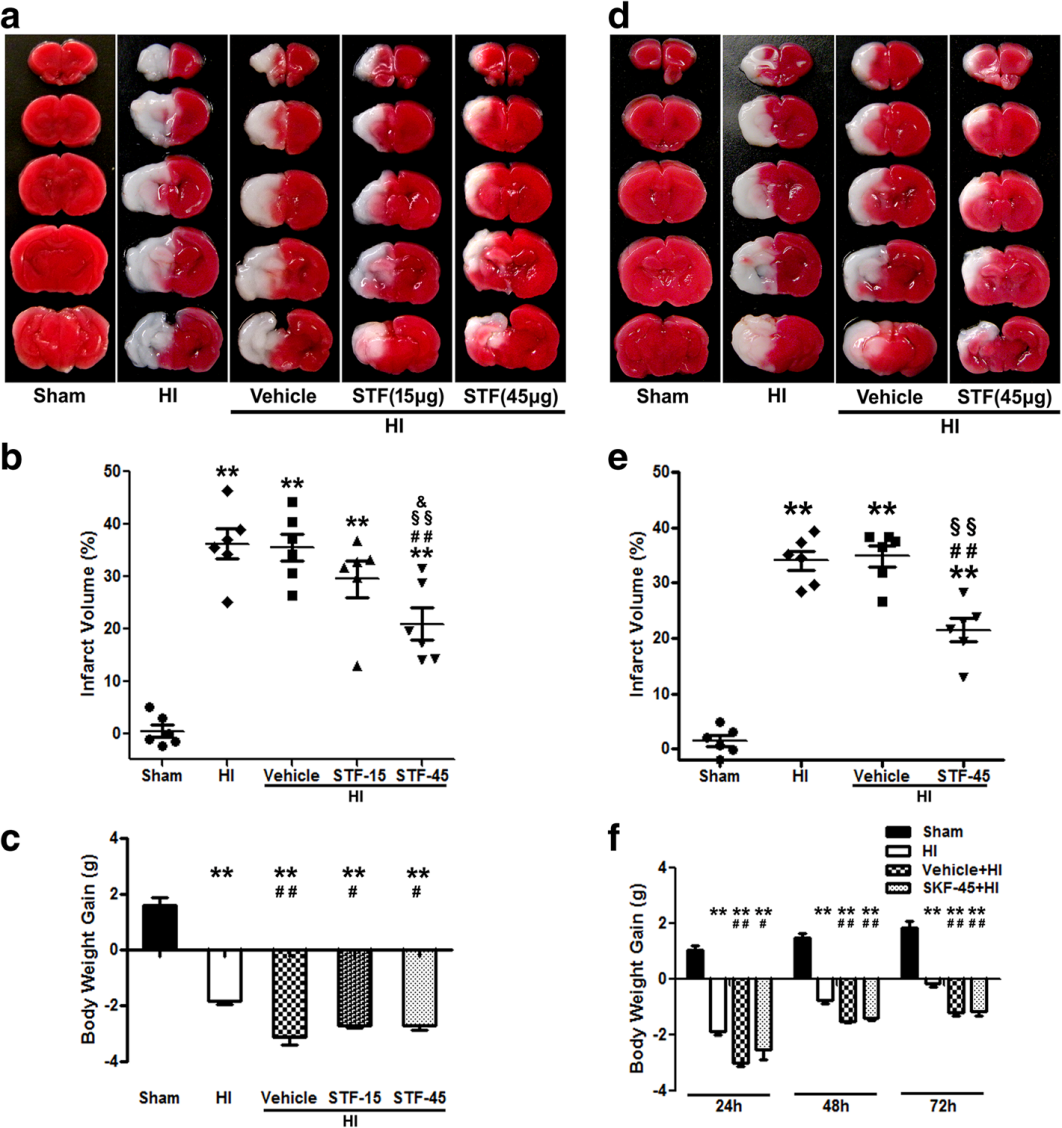
STF-083010, an IRE1α inhibitor, reduced brain infarction at 24 and 72 h post HI**. a**–**c** Representative photographs of TTC-stained coronal brain sections (**a**), quantitative analysis of infarct volume (**b**), and body weight gain (**c**) with single-dose treatment at 24 h post HI. **d**–**f**. Representative photographs of TTC-stained coronal brain sections (**d**), quantitative analysis of infarct volume (**e**), and body weight gain (**f**) with multi-dose treatment at 72 h post HI. Data are represented as mean ± SEM, *n* = 6 in each group. **P < 0.01 compared with sham group, ^##^*P* < 0.01 compared with HI group, ^#^*P* < 0.05 compared with HI group, ^§§^*P* < 0.01 compared with vehicle+HI group, and ^&^*P* < 0.05 compared with STF-15+HI group. STF: STF-083010, an IRE1α RNase-specific inhibitor. STF-15: STF-083010 (15 μg/pup). STF-45: STF-083010 (45 μg/pup)

To evaluate the effect of IRE1α-mediated UPR on brain injury post HI, STF-083010, an IRE1α RNase-specific inhibitor, was intranasally administered at 1 h post HI. Quantitative assessment of TTC-stained sections showed that treatment with the low dose of STF-083010 (15 μg/pup) had an infarct ratio of 29.5% ± 3.5% (*P* > 0.05 vs. HI or vehicle+HI). Administration of high-dose STF-083010 (45 μg/pup) resulted in a significant decrease in brain infarct volume (20.9% ± 3.1%) as compared with the HI (36.2% ± 2.8%) or vehicle+HI (35.5% ± 2.6%) groups (*P* < 0.01) (Fig. 2a, b).

To assess whether multi-dose treatment of IRE1α inhibitor is a more powerful therapeutic strategy than single-dose treatment, STF-083010 (45 μg/pup) was intranasally administered at 1, 24, and 48 h post HI (three total administrations). The results showed that multi-dose STF-083010 treatment significantly reduced infarct volume (21.5% ± 2.1%) at 72 h post HI as compared with the HI (34.0% ± 1.7%) or vehicle+HI (34.9% ± 1.9%) groups (*P* < 0.01) (Fig. 2d, e). However, infarct volume was similar between the single- and multi-dose groups.

On the other hand, multi-dose treatment of STF-083010 or vehicle resulted in worse general well-being since the toxicity of vehicle (cremophor). Body weight of pups was monitored post HI insult as an indicator of their general health. Vehicle and STF groups gained less weight as compared to HI group in each time point (Fig. 2c, f). Moreover, it seems that multi-dose treatment of IRE1α inhibitor or vehicle increased the mortality. The mortality at 72 h post HI insult was 0 (0 of 6) in the HI group, 25% (2 of 8) in the vehicle+HI group, and 14.3% (1 of 7) in the STF-45+HI group. Therefore, the best dose (45 μg/pup) and single-dose administration was chosen to use throughout the duration of the study.

### Physical development was not altered after IRE1α inhibition at 6 weeks post HI

**Fig. 3:**
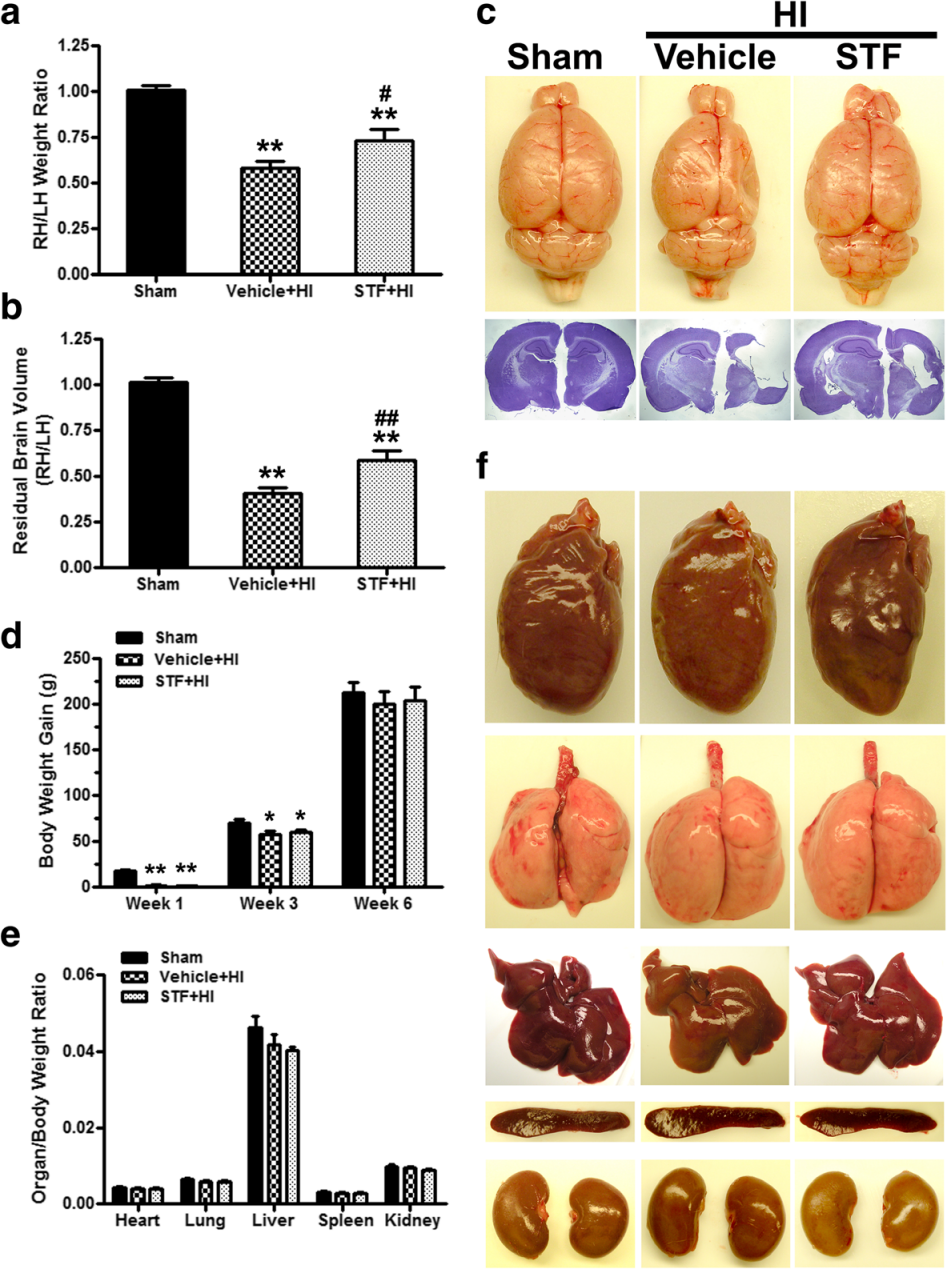
STF-083010 ameliorated brain atrophy and improved physical development at 6 weeks post HI. **a**–**c** Quantification of right (ipsilateral) to left (contralateral) hemispheric (RH/LH) weight ratio showed that brain atrophy was attenuated after treatment (**a**), statistical analyses of residual brain volume showed that treatment significantly attenuated HI induced brain tissue loss (**b**) and top view of the brains and brain slices with Nissl staining at 6 weeks post HI (**c**). **d**–**f** Body weight gain (**d**), quantification of organ (heart, lung, liver, spleen, and kidney) to body weight ratios showed no significant difference between groups (**e**) and representative photographs of organs (**f**). Data are expressed as mean ± SEM, *n* = 8 in sham or vehicle+HI groups and 9 in STF + HI group. **P < 0.01 compared with sham group, *P < 0.05 compared with sham group, ^##^*P* < 0.01 compared with vehicle+HI group, and ^#^*P* < 0.05 compared with vehicle+HI group

Somatic growth retardation starting post HI insult is a common result [53, 54]. Accordingly, vehicle+HI group gained significantly less weight than sham group after 1 week (1.44 ± 0.56 g vs. 17.22 ± 0.53 g, *P* < 0.01) and 3 weeks (56.50 ± 4.15 g vs. 69.81 ± 4.02 g, *P* < 0.05) post HI; STF-083010 treatment did not improve weight gain as compared to vehicle at both time-points (1 weeks: 0.61 ± 0.58 g; 3 weeks: 59.58 ± 1.99 g, *P* > 0.05 vs. vehicle+HI). Vehicle (198.99 ± 14.53 g) and STF-083010 (203.24 ± 14.64 g) treatment pups, however, caught up in weight since the amount of weight gained did not significantly differ from that of sham group (211.83 ± 11.94 g) after 6 weeks post HI (Fig. 3d).

The effects of IRE1α inhibition were explored across multiple organ systems. When compared to sham group, vehicle+HI and STF-083010+HI groups did not show significant differences in heart-, lung-, liver-, spleen-, and kidney-to-body weight ratio (Fig. 3e, f).

### IRE1α inhibition reduced brain atrophy at 6 weeks post HI

Because hemispheric weight loss is an established estimate of brain damage in the HI model [55], the brain atrophy was measured after neurobehavioral tests as previously described [34]. HI insult resulted in severe brain atrophy of the lesion hemispheres at 6 weeks post-HI, marked by a reduction in right to left hemispheric weight ratio (1.01 ± 0.02 vs. 0.58 ± 0.04, *P* < 0.01); it was remarkably attenuated after STF-083010 treatment (0.73 ± 0.06, *P* < 0.05 vs. vehicle+HI) at 6 weeks post-insult (Fig. 3a, c).

Nissl-stained coronal brain sections at 6 weeks post-injury showed brain atrophy in vehicle+HI group at 6 weeks post HI, as demonstrated by vacuolization, neuronal loss, and tissue breakdown (Fig. 3c). Morphology quantification studies of brain volume loss confirmed the statistical significance between sham and vehicle+HI groups (1.01 ± 0.02 vs. 0.41 ± 0.03, *P* < 0.01). STF-083010 treatment significantly reduced the brain tissue loss (0.59 ± 0.05, *P* < 0.01 vs. vehicle+HI, Fig. 3b).

### IRE1α inhibition improved long-term neurological function at 5 or 6 weeks post HI

**Fig. 4:**
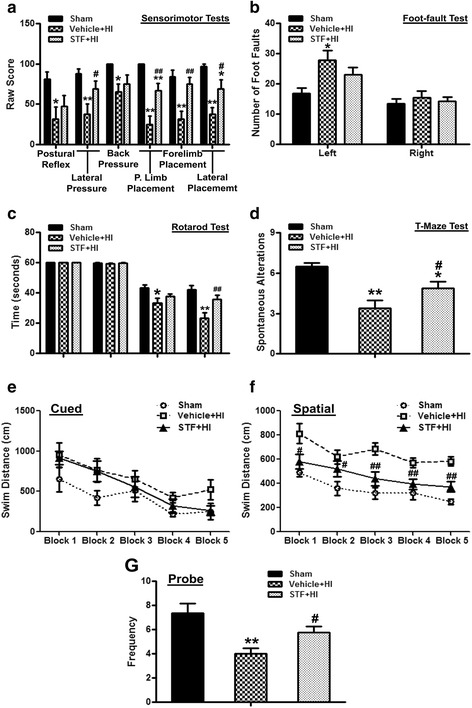
STF-083010 improved long-term neurobehavioral function at 5 and 6 weeks post HI**.** All animals in the HI group exhibited severe neurobehavioral impairments in the sensorimotor tests (**a**), foot fault test (**b**), rotarod test (**c**), T-maze test (**d**), and Morris water maze test (**e**–**g**). STF-083010 treatment significantly improved neurological outcomes in the above tests except for foot fault test (**b**). Data are expressed as mean ± SEM, n = 8 in sham or vehicle+HI groups and 9 in STF+HI group. **P < 0.01 compared with sham group, *P < 0.01 compared with sham group, ^##^*P* < 0.01 compared with vehicle+HI group, ^#^*P* < 0.05 compared with vehicle+HI group

Motor, cognitive, and behavioral deficits may be lifetime consequences of perinatal stroke [56]. Several neurological behavior tests were used to evaluate the role of IRE1α inhibition on long-term neurological function (Fig. 4).

In all sensorimotor tests, sham group operated animals performed well while the vehicle+HI group performed remarkably worse when compared to sham (postural reflex, back pressure: *P* < 0.05; lateral pressure, proprioceptive limb placing, forelimb placement, lateral placement: *P* < 0.01 vs. sham, Fig. 4a). In lateral pressure, proprioceptive limb placement, forelimb placement test, lateral lime placement tests, STF-083010 treatment group significantly improved these deficits across tasks (lateral pressure, lateral placement: *P* < 0.05 vs. vehicle+HI; proprioceptive limb placing, forelimb placement: *P* < 0.01 vs. vehicle+HI, Fig. 4a).

In both accelerating velocity rotarod tests (5 and 10 rpm), animals in vehicle+HI group had a significantly shorter latency to fall compared to the sham group at 6 weeks post HI (5 rpm: 33.28 ± 3.24 s vs. 43.39 ± 2.00 s, *P* < 0.05; 10 rpm: 23.18 ± 3.81 s vs. 42.06 ± 2.71 s, *P* < 0.01, Fig. 4c). Although STF-083010 treatment appeared to improve the HI-induced deficits, only in accelerating 10 rpm tests a significant difference was observed between treatment and vehicle groups (35.85 ± 2.81 s vs. 23.19 ± 3.81 s, *P* < 0.01, Fig. 4c).

Animals in the vehicle+HI group had significantly more foot faults with their left fore- and hind limbs, which is contralateral to the brain injury site, compared with the sham group (27.88 ± 3.23 vs. 16.75 ± 1.89, *P* < 0.05, Fig. 4b). STF-083010 treatment attenuated the increased number of foot faults in HI injury rats, but the difference was not significant between the treatment and vehicle groups (*P* > 0.05, Fig. 4b).

In the T-maze test, the vehicle+HI group demonstrated a significant reduction in exploratory behavior and short-term memory when compared to sham (3.38 ± 0.60 vs. 6.5 ± 0.27, *P* < 0.01, Fig. 4d). STF-083010 significantly improved this neurobehavioral deficit (4.89 ± 0.45, *P* < 0.05 vs. vehicle+HI, Fig. 4d).

No significant difference in the swimming distance (from releasing point to reach the platform on the cued and spatial maze) among all the groups in the cued trials (*P* > 0.05, Fig. 4e). In the spatial maze test, the vehicle+HI group had significantly worse spatial learning than the sham animals and a longer swimming distance (*P* < 0.05, Fig. 4f). STF-083010 treatment group traveled remarkably a shorter distance to find the platform (*P* < 0.05, Fig. 4f). In the probe trials, the frequency of the crossing target quadrant was recorded. The crossing frequency was significantly reduced in the vehicle+HI group (4.00 ± 0.46 vs. 7.38 ± 0.78, *P* < 0.01, Fig. 4g), and STF-083010 treatment increased the frequency (5.78 ± 0.49, *P* < 0.05 vs. vehicle+HI, Fig. 4g).

### IRE1α inhibition upregulated miR-17-5p expression at 6 h post HI

**Fig. 5:**
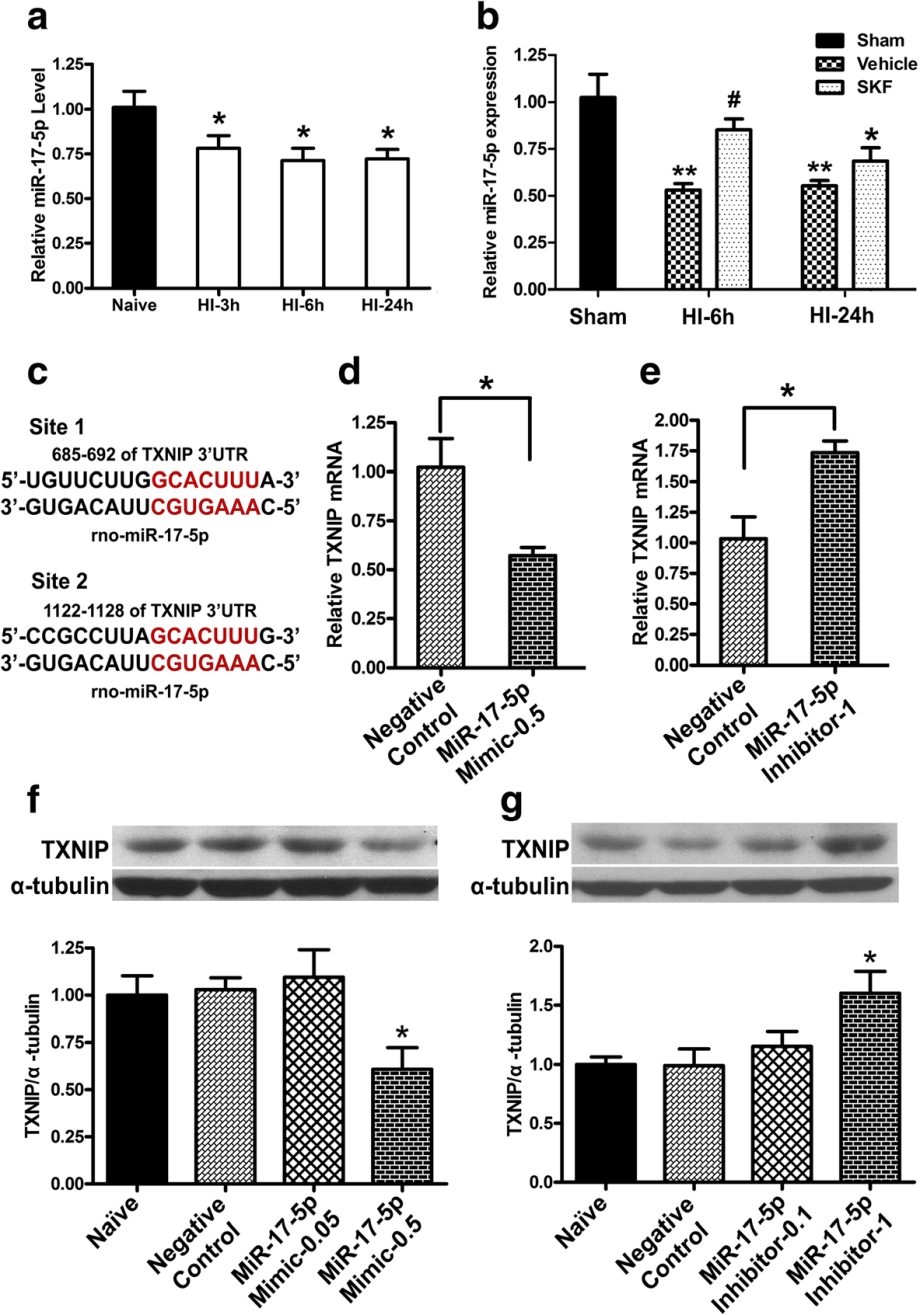
IRE1α inhibition upregulated miR-17-5p expression post HI and. TXNIP is a target of miR-17-5p. **a** qPCR results showed the down-regulation of miR-17-5p in HI compared with naive group. (*n* = 4, *P < 0.05 compared with naive group). **b** q-PCR results showed IRE1α inhibition upregulated mir-17-5p expression at 6 h after HI. (*n* = 4, **P < 0.01 compared with sham group, *P < 0.05 compared with sham group, and ^#^*P* < 0.05 compared with vehicle+HI group). **c** Sequence alignment showed putative miR-17-5p binding sites within the 3′-UTR of the TXNIP mRNA in rats. **d**, **f** The expression levels of TXNIP mRNA (**d**, *n* = 3) and protein (**f**, *n* = 4, *P < 0.05 compared with negative control or naive group) were reduced at 48 h after administration of miR-17-5p mimic. miR-17-5p mimic-0.05 or 0.5: Syn-rno-miR-17-5p miScript miRNA mimic (0.05 or 0.5 nmol/pup). **e**, **g** The expression levels of TXNIP mRNA (**e**, *n* = 3) and protein (**g**, *n* = 4, *P < 0.05 compared with negative control or naive group) were increased at 48 h after administration of miR-17-5p inhibitor. miR-17-5p inhibitor-0.1 or 1: Anti-rno-miR-17-5p miScript miRNA inhibitor (0.1 or 1 nmol/pup)

To explore the mechanistic bases of IRE1α-mediated HI brain injury, we turned our attention to miR-17-5p, a candidate target of IRE1α’s endonuclease activity. Brain tissue was harvested at 3, 6, and 24 h post HI, and expression levels of miR-17-5p were detected using qPCR. The endogenous miR-17-5p level in the ipsilateral hemisphere at 3 h post HI injury showed a decrease in expression levels compared to naive and remained at a relatively low level (*P* < 0.05 vs. Naïve) until 24 h post HI (Fig. 5a).

To confirm that IRE1α acts as a negative regulator of miR-17-5p under HI condition, we examined the miR-17-5p expression levels after IRE1α inhibition. STF-083010 treatment upregulated miR-17-5p expression at 6 and 24 h post HI, but statistical significant difference was observed only at the 6 h time point between the treatment and vehicle groups (*P* < 0.05, Fig. 5b).

### TXNIP expression was regulated by miR-17-5p levels

TXNIP has been implicated in programmed cell death in response to ER stress. It has been confirmed that miR-17 plays a critical role on regulation of TXNIP mRNA stability in ER stress of β cells [23, 25]. Interestingly, two binding sites identified in the TXNIP 3′-UTR might be targeted by miR-17 across multiple species, suggesting a possible critical role of miR-17 in regulation of TXNIP mRNA stability. Using computational miRNA target prediction algorithms TargetScan (http://targetscan.org, Release 7.1), we found that two binding sites identified in the TXNIP 3′-UTR might be targeted by miR-17-5p, a mature miR-17 in rats (Fig. 5c). It has been experimentally validated that TXNIP is a target gene for miR-17-5p [25, 57].

The effect of changing miR-17-5p level on TXNIP in vivo was evaluated by injecting either syn-rno-miR-17-5p miScript miRNA Mimic or anti-rno-miR-17-5p miScript miRNA Inhibitor by intracerebroventricular (ICV) infusion. At 48 h after administration of miR-17-5p mimic or inhibitor, the expression levels of TXNIP mRNA and protein were measured. TXNIP levels were remarkably up-regulated by introducing miR-17-5p inhibitor (1 nmol/pup) into pups (Fig. 5e, g); conversely, miR-17-5p mimic (0.5 nmol/pup) reduced baseline levels of TXNIP (Fig. 5d, f).

### IRE1α showed to alter TXNIP levels through miR-17 post HI

**Fig. 6:**
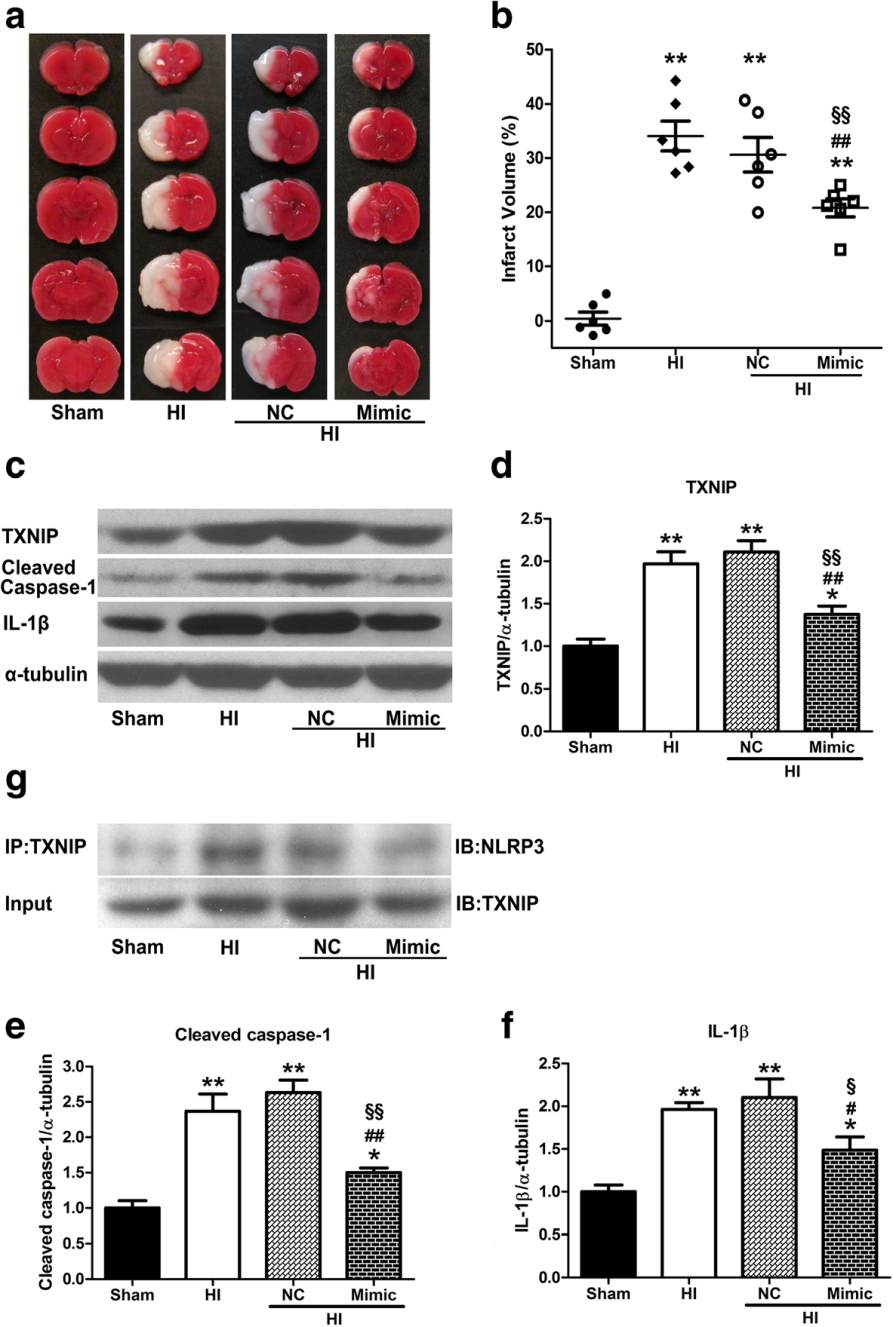
miR-17-5p mimic attenuated infarction, downregulated TXNIP expression, NLRP3 inflammasome activation and IL-1β production at 24 h post HI. **a**, **b** Representative photographs of TTC-stained coronal brain sections (**a**) and quantitative analysis of infarct volume (**b**) at 24 h post HI. **c**–**f** Representative western blotting bands (**c**) and quantification (**d**–**f**) of TXNIP, cleaved caspase-1, and IL-1β expressions. **g** Representative Co-IP assay bands for interaction between NLRP3 and TXNIP. Data are expressed as mean ± SEM, *n* = 6 in each group. **P < 0.01 compared with sham group, *P < 0.05 compared with sham group, ^##^*P* < 0.01 compared with HI group, ^#^*P* < 0.05 compared with HI group, ^§§^*P* < 0.01 compared with NC + HI group, and ^§^*P* < 0.05 compared with NC+HI group. NC negative control

**Fig. 7:**
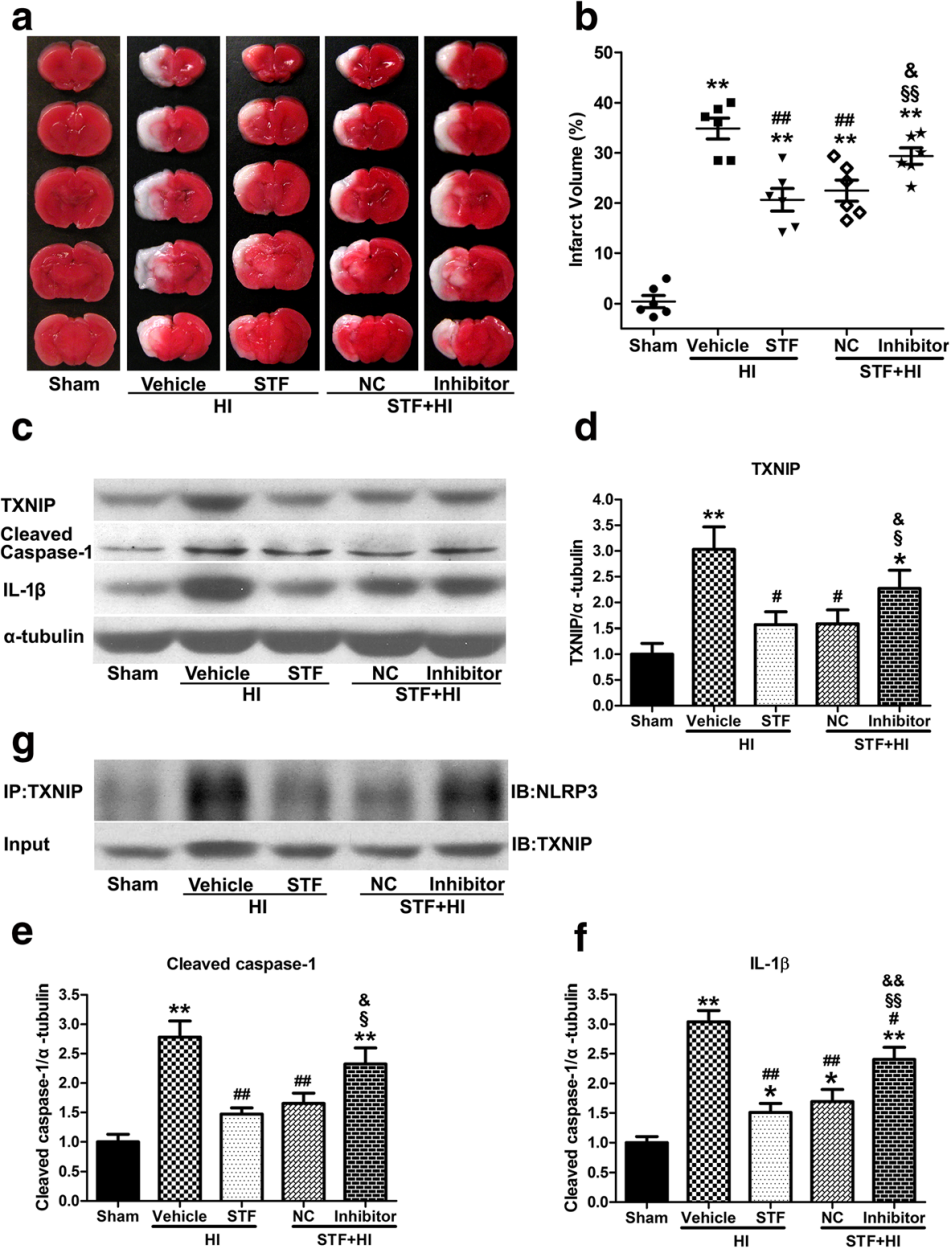
miR-17-5p inhibitor increased infarction, upregulated TXNIP expression, NLRP3 inflammasome activation and IL-1β production at 24 h post HI. **a**, **b** Representative photographs of TTC-stained coronal brain sections (**a**) and quantitative analysis of infarct volume (**b**) at 24 h post HI. **c**–**f** Representative western blotting bands (**c**) and quantification (**d**–**f**) of TXNIP, cleaved caspase-1 and IL-1β expressions. **g** Representative Co-IP assay bands for interaction between NLRP3 and TXNIP. Data are expressed as mean ± SEM, n = 6 in each group. **P < 0.01 compared with sham group, *P < 0.05 compared with sham group, ^##^*P* < 0.01 compared with vehicle+HI group, ^#^*P* < 0.05 compared with vehicle+HI group, ^§§^*P* < 0.01 compared with STF+HI group, ^§^*P* < 0.05 compared with STF+HI group, ^&&^*P* < 0.01 compared with STF+NC+HI group, and ^&^*P* < 0.05 compared with STF+NC+HI group

We next investigated whether the IRE1α regulated TXNIP expression through miR-17 post HI. Either Syn-rno-miR-17-5p miScript miRNA mimic or Anti-rno-miR-17-5p miScript miRNA inhibitor was administered into pups by ICV infusion 2 days before HI. The expression of TXNIP was significantly (*P* < 0.01) increased post HI compared to sham, but miR-17-5pmiRNA mimic-administrated pups showed significantly (*P* < 0.01) reduced expression of TXNIP compared to HI or negative control+HI groups (Fig. 6c, d). STF-083010 treatment downregulated TXNIP expression post HI (*P* < 0.05 vs. vehicle+HI group), while administration of anti-miR-17-5p miRNA inhibitor resulted in higher TXNIP level (*P* < 0.05 vs. STF+HI or STF+negative control+HI groups, Fig. 7c, d).

### Inhibition of IRE1α with STF-083010 exerts its neuroprotective effects via overexpression of miR-17-5p at 24 h post HI

We injected either Syn-miR-17-5p mimic or Anti-miR-17-5pmiRNA inhibitor into pups by ICV infusion two days before HI and measured infarct volume at 24 h post HI. As shown in Fig. 6a, b, pups pretreated with Syn-rno-miR-17-5p miScript miRNA Mimic showed reduced infarct size in the brain (*P* < 0.01 vs. HI or negative control+HI group, Fig. 6a, b). STF-083010 treatment ameliorates brain injury post HI, however the therapeutic effect was reversed by Anti-rno-miR-17-5p miScript miRNA inhibitor (*P* < 0.05 vs. STF+negative control+HI group, Fig. 7a, b).

### miR-17 played a role in IRE1a-mediated NLRP3 inflammasome activation and IL-1β production at 24 h post HI

It has previously been known that TXNIP, as a binding partner to NLRP3, is essential for NLRP3 inflammasome activation and IL-1β production under oxidative stress and ER stress, raising the possibility that NLRP3 inflammasome activation is the downstream event of IRE1α-induced TXNIP upregulation through miR-17 after HI. To experimentally establish this hypothesis, we first detected whether intervention of miR-17 expression changed IRE1α-induced TXNIP-NLRP3 binding by co-immunoprecipitation (Co-IP) studies. Pups in HI group displayed robust amounts of NLRP3 protein pulled down together with TXNIP compared with sham, however, miR-17-5p miRNA mimic pretreatment reduced TXNIP-NLRP3 binding (Fig. 6g). STF-083010 treatment group showed less TXNIP protein binding with NLRP3, whereas administration of anti-miR-17-5p miRNA Inhibitor reversed this effect (Fig. 7g).

Cleaved caspase-1 and IL-1β expression levels were measured to evaluate the effects of NLRP3 inflammasome activation. miR-17-5p miRNA mimic pretreatment suppressed elevated cleavage of caspase-1 (*P* < 0.01 vs. negative control+HI group, Fig. 6c, e) and downregulation of IL-1β release after HI injury (*P* < 0.05 vs. negative control+HI group, Fig. 6c, f). IRE1α inhibition prevented increase in cleaved caspase-1 (*P* < 0.01 vs. vehicle+HI group, Fig. 7c, e) and IL-1β (*P* < 0.01 vs. vehicle+HI group, Fig. 7c, f) expression induced by HI injury. Anti-miR-17-5p miRNA inhibitor also attenuated this effect (Fig. 7c, f).

## Discussion

Neonatal hypoxic–ischemic encephalopathy (HIE) is a devastating condition that results in lifelong disabilities. It is caused due to hypoxemia or reduced cerebral blood flow and underlies much of the morbidity (rate up to 60%) and mortality (rate up to 50%). Neonatal hypoxic–ischemic (HI) brain injury affects 1–4 infants per 1000 births and leads to pulmonary immaturity, respiratory distress syndrome, hypercapnia, hypoperfusion, seizures, and long-term cognitive and behavioral deficits. Most of the current treatments are focused mainly on supportive care and prevention of HI complications, but despite that, high rates of morbidity and mortality remain. Hence, there is a need to peruse alternative strategies and therapies.

One of the main pathologies encountered after HI is endoplasmic reticulum (ER) stress. ER stress is a condition that causes an imbalance of ER homeostasis and accumulation of unfolded/misfolded proteins in the ER lumen induced by several physiological or pathological perturbations at the cellular level, such as hypoxia, acidosis, and calcium depletion. Under remediable ER stress, a series of adaptive mechanisms known as the unfolded protein response (UPR) get activated to emit pro-survival signals and combat ER stress. However, if the ER stress is irreversible, the UPR is not sufficient to restore ER homeostasis and cell death occurs.

IRE1α, one of the three ER-transmembrane protein receptors (PERK, IRE1, and ATF-6), initiating UPR signaling pathways, is identified as a cell fate executor under ER stress. IRE1 contains two enzymatic activities, a kinase and an endoribonuclease (RNase) [58]. During remediable ER stress, IRE1α kinase domain is autophosphorylated and consequently activates the RNase activity to cleave X-box-binding protein 1(XBP1) mRNA at specific sites to excise an intron. This splicing event produces a potent transcription factor called XBP1s whose target genes enhance ER protein folding capacity and quality control and promote adaptation [59–61]. Under irremediable ER stress, IRE1α ribonuclease activity becomes less discriminated and triggers IRE1-dependent decay (RIDD) of multiple substrates, including multiple miRNAs. Although the significance of RIDD targets is not completely understood, some RIDD events are critical for apoptosis [62, 63].

In a recent study Carloni et al. assessed the IRE1 pathway by the splicing of XBP1 mRNA after HI [64]. The spliced Xbp-1 was detectable in the ipsilateral hemispheres 2 h after the insult, but was not observed 24 h post HI. In this study, we demonstrated a temporal profile of IRE1α expression level in the neonatal HI model. Phosphorylated IRE1α level in the ipsilateral hemisphere was upregulated at 0~ 6 h and return to the baseline at 12 h post HI. This is consistent with Carloni’s data. It has been reported that STF-083010, a new IRE1α RNase-specific inhibitor, remarkably decreased the apoptotic ratio in a post-traumatic stress disorder model by attenuating activation of the IRE1α apoptosis pathway. To further confirm whether IR1α promoted neuron death post HI, STF-083010 was administrated in the neonatal HI model. STF-083010 treatment showed to significantly decrease infarct volumes at 24 and 72 h, improved long-term neurological impairments, as well as attenuated brain atrophy at 5 and 6 weeks post HI. These results suggest that IRE1α activation is involved in brain injury after neonatal HI.

Currently, microRNA that generally suppress gene expression, have emerged as key regulators of ER homeostasis and important players in UPR signaling pathway. miR-17 is a member of the miR-17/92 cluster, one of so far, the best-studied microRNA clusters that codes six mature miRNAs: miR-17, miR-18a, miR-19a, miR-20a, miR-19b-1, and miR-92a-1. Members of this cluster are expressed in a variety of tissues and carry out essential functions both in normal development and in diseases. Most functional studies of the miR-17/92 cluster focused on tumorigenesis, in which the cluster promotes proliferation and survival of tumor cells. It is also required for inducing proliferation of multiple cells in individual development, such as cardiomyocytes, neural stem cells, lung epithelium and lymphocyte [65–68]. Among the six members of miR-17/92 cluster, miR-17 is expressed ubiquitously and highly in all tissues detected, pointing to a generally high significance of this miRNA. There is a widespread overexpression of miR-17 in diverse tumor subtypes including both hematopoietic and solid tumors [69–73]. In addition, miR-17 plays a role in neurodegenerative diseases including Alzheimer’s disease and multiple sclerosis [74, 75].

Recently, it has been found that miR-17 could inhibit hypoxia-induced apoptosis in the kidneys, hearts, and pulmonary artery smooth muscle cells [76–79]. However, there are some divergences of the role of miR-17 in hypoxia/ischemia-induced injuries presented by other research groups. Li et al. found that miR-17 overexpression can upregulate autophagy to aggravate hepatic ischemia reperfusion injury [80]. Du et al. Showed that miR-17 promoted cardiomyocyte apoptosis in response to ischemia followed by reperfusion [81]. Furthermore, it has been reported that miR-17 might be a substrate of RE1-dependent decay (RIDD). Upton et al. found sustained IRE1α RNase activation caused rapid decay of select microRNAs (miRs − 17, −34a, − 96, −125b) [62]. Moreover, Lerner et al. also report IRE1α mediated destabilization of miR-17 in β cells [25]. Thus, based on the above studies, we hypothesized that HI induced activation of IRE1 can degenerate miR-17 and exacerbate brain injury. Consistent with this notion, we found that level of miR-17-5p expression in the ipsilateral hemisphere reduced up to 24 h post HI insult and IRE1 RNase inhibition could rescue the miR-17-5p level drop that occurred at 6 h post HI. These data suggest that miR-17-5p might be a potential effector in the IRE1-induced UPR pathway after HI. A previous study indicated that serum miR-17-5p expression was elevated after acute ischemic stroke in the human adult. This considerable discrepancy is probably due to expressions and functions of miR-17/92 cluster depend on various contexts, cellular type, species, model system, and age.

It is known that neuroinflammation plays a principal role in neonatal HI brain damage. Microglial activation and aggregation are pathological markers for HI. Activated microglia produce inflammatory mediators which cause oligodendrocyte death, axonal degeneration and disruption of the immature BBB. Thioredoxin-interacting protein (TXNIP) is a binding partner of reduced thioredoxin (TRX) and functions as a negative regulator of the TRX reductase activity. TXNIP dissociates from TRX after oxidation of TRX by ROS, which allows TXNIP to bind with NLRP3. Interaction between TXNIP and NLRP3 was pivotal for NLRP3 inflammasome activation, and consequent caspase-1 cleavage and IL-1β secretion [19]. Therefore, TXNIP have been thought as a bridge linking oxidative stress and inflammation.

Data from bioinformatic analysis showed the TXNIP 3′-UTR has two conserved binding sites for miR-17. It has been experimentally confirmed that TXNIP acts as a direct target gene for miR-17 in β cells and senescent fibroblasts [25, 57]. Oxidative stress disrupts ER homeostasis and then activates the IER1-induced UPR. Hyperactivated IRE1α displays relaxed-specific RNase activity, initiating RIDD to degenerate miR-17, the TXNIP destabilizing microRNA. MiR-17 level drop increases TXNIP mRNA stability through post-transcriptional regulation. Increased TXNIP combines with NLRP3 to active the inflammasome, subsequently cell fate is switched from an adaptive UPR and cell survival to a terminal UPR and cell death.

Based on the above findings, we may reasonably speculate that the level of TXNIP might be regulated by IRE1-mediated miR-17 decay and then control activation of NLRP3 inflammasome and release of inflammatory mediator after neonatal HI. Consistent with this notion, we found that TXNIP mRNA and protein levels were downregulated by administrating miR-17-5p mimic, whereas anti-miR-17-5p inhibitor upregulated TXNIP expression in the neonatal HI model. Furthermore, miR-17-5p mimic also inhibited NLRP3 binding to TXNIP and prevented NLRP3 inflammasome formation and activation, featuring with caspase-1 cleavage and IL-1β production. Upregulated miR-17-5p provided neuroprotective effect on the neonatal HI pups. Conversely, anti-miR-17-5p inhibitor reversed IRE1 inhibition-induced decrease in NIRP3-TXNIP combination and inflammasome activation, as well as exacerbated brain injury after HI.

## Conclusion

In conclusion, these findings elucidate a novel molecular mechanism mediating HI brain injury, which may be new potential therapeutic target after neonatal HI. We provided direct evidence that IRE1α-induced UPR pathway may contribute to inflammatory activation and brain injury following neonatal HI. IRE1α activation, through degenerating miR-17-5p, stabilized TXNIP mRNA and amplified TXNIP level to activate NLRP3 inflammasome and deteriorate brain injury. This study revealed IRE1α inhibition as a new neuroprotective mechanism and has prospective clinical implications in preventing and treating HI brain injury.

## Additional file

Additional file 1: **Table S1.** STF-083010 reduced brain infarction at 24 after HI (raw data of quantitative analysis of infarct volume). **Table S2.** STF-083010 reduced brain infarction at 72 after HI (raw data of quantitative analysis of infarct volume). **Table S3.** miR-17-5p mimic attenuated brain infarction after HI (raw data of quantitative analysis of infarct volume). **Table S4.** miR-17-5p inhibitor reversed the effect of IRE1a inhibition on brain infarction after HI (raw data of quantitative analysis of infarct volume). (DOCX 19 kb)

## Abbreviations

ATF6: Activating transcription factor 6
BBB: Blood–brain barrier
Co-IP: Co-immunoprecipitation
ER: Endoplasmic reticulum
HI: Hypoxia–ischemia
IRE1: Inositol-requiring enzyme 1
miR-17: MicroRNA-17
NLRP3: Nod-like receptor protein 3
PERK: Protein kinase-like endoplasmic reticulum kinase
RIDD: IRE1-dependent decay
RT-qPCR: Reverse transcription quantitative real-time polymerase chain reaction
TRX: Thioredoxin
TXNIP: Thioredoxin-interacting protein
UPR: Unfolded protein response
XBP1: X-box-binding protein 1
