# Supplementary material for: IRE1α inhibition decreased TXNIP/NLRP3 inflammasome activation through miR-17-5p after neonatal hypoxic–ischemic brain injury in rats
Source: J Neuroinflammation. 2018 Feb 2;15:32. doi: 10.1186/s12974-018-1077-9 (PMC5797348; doi:10.1186/s12974-018-1077-9)
Supplement: Additional file 1: — Table S1. STF-083010 reduced brain infarction at 24 after HI (raw data of quantitative analysis of infarct volume). Table S2. STF-083010 reduced brain infarction at 72 after HI (raw data of quantitative analysis of infarct volume). Table S3. miR-17-5p mimic attenuated brain infarction after HI (raw data of quantitative analysis of infarct volume). Table S4. miR-17-5p inhibitor reversed the effect of IRE1a inhibition on brain infarction after HI (raw data of quantitative analysis of infarct volume). (DOCX 19 kb) [file 12974_2018_1077_MOESM1_ESM.docx]

**Additional file**

**Additional file 1: Table S1 STF-083010 reduced brain infarction at 24 after HI (raw data of quantitative analysis of infarct volume)**

|  | **Sham** | | **HI** | | **Vehicle + HI** | | **SKF-15+HI** | | **SKF-45+HI** | |
| --- | --- | --- | --- | --- | --- | --- | --- | --- | --- | --- |
|  | **Total area of CH** | **Area of uninfarcted area of IH** | **Total area of CH** | **Area of uninfarcted area of IH** | **Total area of CH** | **Area of uninfarcted area of IH** | **Total area of CH** | **Area of uninfarcted area of IH** | **Total area of CH** | **Area of uninfarcted area of IH** |
| **1** | 2.528 | 2.588 | 1.688 | 0.531 | 2.484 | 0.787 | 1.712 | 1.271 | 2.739 | 1.009 |
| **2** | 2.892 | 2.721 | 1.828 | 0.407 | 1.712 | 0.199 | 1.772 | 0.652 | 2.736 | 1.957 |
| **3** | 1.983 | 2.078 | 1.806 | 0.127 | 2.018 | 0.517 | 2.155 | 0.874 | 1.895 | 1.361 |
| **4** | 2.635 | 2.372 | 2.44 | 1.216 | 1.625 | 0.628 | 1.8 | 0.606 | 1.918 | 1.261 |
| **5** | 1.887 | 1.947 | 1.95 | 0.502 | 1.609 | 0.305 | 1.746 | 0.459 | 1.817 | 0.777 |
| **6** | 2.065 | 2.067 | 2.083 | 0.598 | 1.921 | 0.904 | 1.902 | 0.655 | 2.086 | 1.269 |

CH: Contralateral hemisphere

IH: Ipsilateral hemisphere

**Additional file 1: Table S2 STF-083010 reduced brain infarction at 72 after HI (raw data of quantitative analysis of infarct volume)**

|  | **Sham** | | **HI** | | **Vehicle+HI** | | **SKF-45+HI** | |
| --- | --- | --- | --- | --- | --- | --- | --- | --- |
|  | **Total area of CH** | **Area of uninfarcted area of IH** | **Total area of CH** | **Area of uninfarcted area of IH** | **Total area of CH** | **Area of uninfarcted area of IH** | **Total area of CH** | **Area of uninfarcted area of IH** |
| **1** | 2.589 | 2.554 | 2.203 | 0.953 | 1.905 | 0.477 | 2.272 | 0.987 |
| **2** | 2.834 | 2.664 | 1.974 | 0.803 | 1.744 | 0.409 | 1.786 | 1.095 |
| **3** | 1.965 | 2.041 | 2.451 | 0.764 | 1.97 | 0.456 | 2.024 | 1.146 |
| **4** | 2.759 | 2.766 | 2.407 | 0.517 | 1.719 | 0.623 | 1.789 | 1.327 |
| **5** | 2.318 | 2.221 | 2.151 | 0.548 | 2.239 | 1.043 | 1.651 | 0.89 |
| **6** | 2.548 | 2.303 | 2.126 | 0.632 | 2.106 | 0.568 | 2.063 | 1.083 |

CH: Contralateral hemisphere

IH: Ipsilateral hemisphere

**Additional file 1: Table S3 miR-17-5p mimic attenuated brain infarction after HI (raw data of quantitative analysis of infarct volume)**

|  | **Sham** | | **HI** | | **NC+HI** | | **Mimic+HI** | |
| --- | --- | --- | --- | --- | --- | --- | --- | --- |
|  | **Total area of CH** | **Area of uninfarcted area of IH** | **Total area of CH** | **Area of uninfarcted area of IH** | **Total area of CH** | **Area of uninfarcted area of IH** | **Total area of CH** | **Area of uninfarcted area of IH** |
| **1** | 2.528 | 2.588 | 3.212 | 1.463 | 2.394 | 1.172 | 2.408 | 1.299 |
| **2** | 2.892 | 2.721 | 1.954 | 0.225 | 2.211 | 1.329 | 2.125 | 1.249 |
| **3** | 1.983 | 2.078 | 2.09 | 0.698 | 2.069 | 0.478 | 2.319 | 1.336 |
| **4** | 2.635 | 2.372 | 2.042 | 0.764 | 2.07 | 0.89 | 2.044 | 1.509 |
| **5** | 1.887 | 1.947 | 1.971 | 0.393 | 2.223 | 0.416 | 2.163 | 1.21 |
| **6** | 2.065 | 2.067 | 2.063 | 0.892 | 2.009 | 0.776 | 2.813 | 1.406 |

CH: Contralateral hemisphere

IH: Ipsilateral hemisphere

**Table 4 miR-17-5p inhibitor reversed the effect of IRE1a inhibition on brain infarction after HI (raw data of quantitative analysis of infarct volume)**

|  | **Sham** | | **Vehicle+HI** | | **SKF+HI** | | **SKF+NC+HI** | | **SKF+inhibitor+HI** | |
| --- | --- | --- | --- | --- | --- | --- | --- | --- | --- | --- |
|  | **Total area of CH** | **Area of uninfarcted area of IH** | **Total area of CH** | **Area of uninfarcted area of IH** | **Total area of CH** | **Area of uninfarcted area of IH** | **Total area of CH** | **Area of uninfarcted area of IH** | **Total area of CH** | **Area of uninfarcted area of IH** |
| **1** | 2.528 | 2.588 | 2.946 | 1.266 | 2.497 | 1.43 | 2.445 | 1.253 | 1.899 | 0.853 |
| **2** | 2.892 | 2.721 | 1.969 | 0.503 | 2.149 | 0.903 | 2.42 | 1.116 | 2.153 | 0.72 |
| **3** | 1.983 | 2.078 | 1.465 | 0.629 | 2.228 | 1.159 | 2.163 | 1.379 | 2.108 | 0.838 |
| **4** | 2.635 | 2.372 | 1.952 | 0.439 | 2.331 | 1.672 | 2.158 | 1.445 | 2.107 | 1.129 |
| **5** | 1.887 | 1.947 | 1.961 | 0.389 | 2.046 | 1.426 | 2.19 | 1.335 | 2.015 | 0.646 |
| **6** | 2.065 | 2.067 | 2.325 | 0.644 | 2.2 | 1.308 | 2.206 | 0.91 | 2.158 | 0.948 |

CH: Contralateral hemisphere

IH: Ipsilateral hemisphere
